# Workstyle Reform for Japanese Doctors Did Not Improve Surgical Productivity in the Long Term

**DOI:** 10.31662/jmaj.2025-0575

**Published:** 2026-07-24

**Authors:** Yoshinori Nakata, Yuichi Watanabe, Akihiko Ozaki

**Affiliations:** 1 Teikyo University Medical Information and System Research Center and Teikyo University Graduate School of Public Health, Tokyo, Japan; 2 Institute of Developing Economies, Japan External Trade Organization, Tokyo, Japan; 3 Department of Breast and Thyroid Surgery, Jyoban Hospital, Fukushima, Japan

**Keywords:** workstyle reform, productivity, Malmquist index, surgery

## Abstract

**Introduction:**

This study aimed to determine the long-term effects of workstyle reform for doctors in Japan on surgical productivity changes using the Malmquist index (MI) model. We hypothesized that this reform would significantly increase surgical total factor productivity in the long term.

**Methods:**

We conducted a retrospective observational study at a university hospital covering the period from June 1 to September 30 during the years 2022 to 2025. A nonradial, nonoriented MI model with variable returns to scale was applied. The decision-making unit (DMU) was defined as the senior surgeon responsible for each procedure. Inputs included (1) the number of assisting surgeons and (2) operative duration from skin incision to closure. The output was defined as the surgical fee per procedure. Changes observed between 2022 and 2023 were categorized as “prereform,” whereas those between 2024 and 2025 were categorized as “postreform.” All inputs and outputs were aggregated for each DMU. The primary outcomes were productivity changes, and the secondary outcomes were efficiency and technical changes between the pre- and postreform periods.

**Results:**

A total of 5,415 surgical procedures performed by 62 surgeons were analyzed. There was no statistically significant difference in overall productivity change between the pre- and postreform periods. However, there was a significant increase in efficiency change and a significant decrease in technical change between the pre- and postreform periods.

**Conclusions:**

No evidence was found to suggest that the workstyle reform for doctors in Japan increased their surgical total factor productivity in the long term. The counterbalancing effects of efficiency and technical changes resulted in no change in overall productivity.

## Introduction

### Background/rationale

The reform of physicians’ workstyles represents a key component of Japan’s labor policy, primarily by imposing legal limits on overtime work^(1)^. Although the workstyle reform law for general workers took effect on April 1, 2019, its physician-specific regulations were implemented nationwide on April 1, 2024^(2)^. Doctors in Japan have historically worked exceptionally long hours, a practice that has supported the healthcare system despite challenges posed by workforce shortages and population aging. However, excessive working hours have also contributed to physician burnout and an increased risk of medical errors ^(3), (4)^.

Extended working hours among physicians have had international repercussions, prompting large-scale protests and strikes by junior doctors in countries such as the United Kingdom and South Korea, thereby disrupting healthcare delivery ^(5)^. In Japan, younger physicians increasingly avoid surgical specialties, resulting in stagnant growth in the number of surgeons and obstetricians ^(1)^.

The workstyle reform is expected to enhance productivity by improving physician health and reducing medical errors, thereby sustaining healthcare quality under constrained labor conditions. Nonetheless, our previous research found no evidence of a short-term improvement in surgical total factor productivity following the reform ^(6)^. This study therefore focuses on evaluating its long-term effects.

Advances in objective methods for assessing efficiency and productivity have accelerated since 2000 across economics, management science, and engineering ^(7), (8)^. Data envelopment analysis (DEA) is widely used for efficiency assessment, while the Malmquist index (MI) model extends DEA to longitudinal analyses by measuring productivity changes over time. The MI model decomposes productivity change into efficiency change (EC) and technical change (TC), thereby clarifying the underlying sources of variation ^(9)^.

### Objectives

This study was conducted to determine the long-term effects of this workstyle reform on surgical productivity changes using the MI model; it was also performed to evaluate the long-term impacts of the reform on surgical EC and TC. We hypothesized that the workstyle reform for doctors would significantly increase their surgical total factor productivity in the long term.

## Materials and Methods

### Study design

This retrospective observational study was approved by the Teikyo University Institutional Review Board (approval number: 12-030-4). Informed consent was waived due to the study’s retrospective nature. Reporting adhered to the Strengthening the Reporting of Observational Studies in Epidemiology (STROBE) guidelines ^(10)^.

### Setting

The study was carried out at Teikyo University Hospital in metropolitan Tokyo, Japan, a tertiary care institution with 1,152 beds that serves approximately 1 million residents. The hospital performs roughly 9,000 surgical procedures annually across 13 specialties. It is classified under the “A standard” category of the workstyle reform, which imposes the strictest annual overtime limit of fewer than 960 hours per physician ^(11)^.

### Participants

We collected data on all surgical procedures performed in the main operating rooms of Teikyo University Hospital between June 1 and September 30 during the years 2022 to 2025. All data were obtained from the hospital’s electronic medical record system. We chose this time frame because the workstyle reform was implemented on April 1, 2024, and the fee schedule revision started on June 1, 2024. Because of budgetary and time constraints, we could not collect full-year data. The period from June 1 through September 30 in 2024-2025 was the only time window in which the effects of the workstyle reform could be evaluated without the influence of the fee schedule revision ^(12), (13)^. We chose the corresponding period (June 1-September 30) in 2022-2023 as the control period. Our total study period was, therefore, 16 months (4 months in each year for 4 years). We defined in this study the changes from 2022 to 2023 as “prereform” and those from 2024 to 2025 as “postreform.”

Procedures were excluded if they were performed under local anesthesia; involved primarily oral, ophthalmic, or dermatologic surgery; resulted in patient death within 1 month; were not reimbursed under the surgical payment system; or contained incomplete data ^(14), (15)^.

### Variables

The MI quantifies productivity change of a decision-making unit (DMU) between two periods and decomposes it into EC and TC components. EC reflects changes in relative efficiency, whereas TC represents shifts in the technological frontier ^(7), (8), (9)^. Mathematically, the MI is defined as the product of EC and TC and represents total factor productivity change. The productivity change of a DMU between Periods 1 and 2 is expressed as follows ^(9)^:


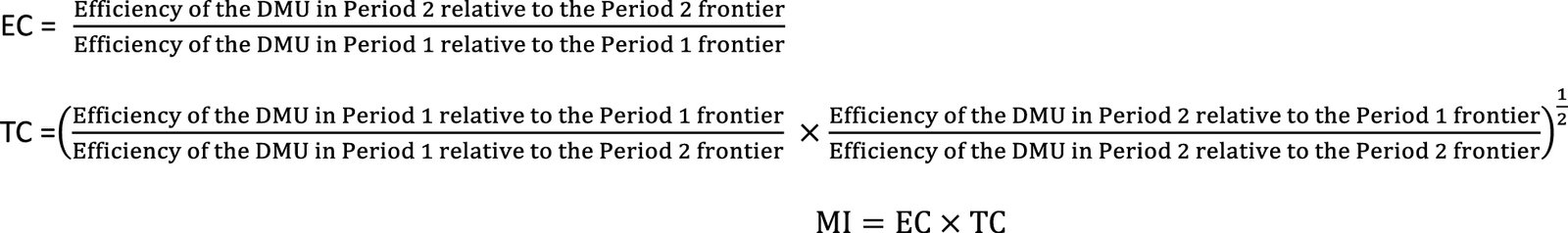


In summary, the MI reflects total factor productivity change of a DMU by capturing both improvements or declines in relative efficiency and shifts in the technological frontier ^(9)^.

### Data sources/measurement

A nonradial, nonoriented MI model with variable returns to scale was applied. The DMU was defined as the senior surgeon responsible for each procedure. We studied the surgeons who performed surgery during the study periods (June 1-September 30) in all 4 years (2022-2025). Inputs included the number of assisting surgeons and operative duration, whereas output was measured as the surgical fee based on Japanese “K codes.” This fee is uniform regardless of surgeon experience (licensed attending surgeon or trainee), number of assistants, or operative duration ^(12), (13)^. Additional fees related to blood transfusions, medications, special insurance medical materials, and anesthesia were excluded. All fees were converted from Japanese yen to US dollars using an exchange rate of ¥150 per dollar to ensure international comparability.

### Bias and study size

By analyzing all eligible procedures performed during the same seasonal window across 4 years, potential seasonal bias was minimized. Given the hospital’s surgical volume, the sample size was considered sufficient to reduce the risk of type II error.

### Quantitative variables

Inputs and outputs were aggregated annually for each DMU. MI, EC, and TC were calculated for the pre- and postreform periods using DEA-Solver-Pro software, version 12.1 (Saitech, Inc., Tokyo, Japan) ^(7), (9)^. The primary outcomes were productivity changes (MI), and the secondary outcomes were EC and TC between 2022 and 2023 and between 2024 and 2025.

All surgeons within the surgical departments were assigned MI, EC, and TC values. Natural logarithms of these indices were calculated to facilitate interpretation as percentage changes ^(16), (17), (18)^. A natural logarithm of the MI greater than, equal to, or less than 0 indicated increased, unchanged, or decreased productivity, respectively. Similarly, positive values of the natural logarithms of EC and TC indicated improvements in efficiency and technology, respectively. The natural logarithm of MI equals the sum of the natural logarithms of EC and TC ^(17), (18)^.

The following 10 surgical specialties were included: cardiovascular surgery, emergency surgery, general surgery, neurosurgery, obstetrics and gynecology, orthopedics, otolaryngology, plastic surgery, thoracic surgery, and urology. For each specialty, the natural logarithms of MI, EC, and TC were calculated for each surgeon, and the mean values and standard errors were determined ^(18)^.

### Statistical methods

Statistical analyses were performed using Excel Statistics Software (Social Survey Research Information Co., Ltd., Tokyo, Japan). Pre- and postreform values were compared using paired t-tests, with statistical significance defined as p < 0.05 ^(19), (20)^.

## Results

### Participants

A total of 5,415 surgical procedures were included in the analysis, comprising 2,781 procedures in the prereform period and 2,634 procedures in the postreform period. These procedures were performed by 62 surgeons.

### Descriptive data and outcome data

The characteristics of surgical procedures in the pre- and postreform periods are summarized in Tables 1 and 2, respectively. The mean number of assisting physicians per procedure was 2.6 in the prereform period and 2.5 in the postreform period. The mean operative duration was 155 minutes per case in the prereform period, with a mean surgical fee of $2,828. In the postreform period, the corresponding values were 160 minutes per case and $2,911.

**Table 1.** Characteristics of Surgery 2022-2023 (Prereform Period).

Specialties	DMUs	Cases	Assistants per case, mean	Operative duration per case (min), mean	Fee per case (US dollars), mean
Cardiovascular surgery	3	327	3.45	173	5,100
Emergency surgery	6	404	2.49	118	2,065
General surgery	13	483	2.42	176	2,622
Neurosurgery	6	124	1.98	235	5,873
Obstetrics and gynecology	7	316	2.13	146	2,230
Orthopedics	11	515	2.77	142	2,215
Otolaryngology	4	115	1.43	123	1,485
Plastic surgery	6	172	2.38	139	1,504
Thoracic surgery	3	132	2.87	183	4,078
Urology	3	193	2.97	157	2,874
All surgical procedures	62	2,781	2.58	155	2,828

Note: The monetary values of surgical fees were converted to US dollars at $1 = ¥150. DMU: decision-making unit.

**Table 2.** Characteristics of Surgery 2024-2025 (Postreform Period).

Specialties	Cases	Assistants per case, mean	Operative duration per case (min), mean	Fee per case (US dollars), mean
Cardiovascular surgery	312	2.93	151	4,516
Emergency surgery	379	2.44	133	2,244
General surgery	438	2.18	192	2,934
Neurosurgery	137	2.61	312	6,228
Obstetrics and gynecology	324	2.19	140	2,226
Orthopedics	459	2.61	140	2,611
Otolaryngology	110	1.44	125	1,435
Plastic surgery	168	2.06	129	1,518
Thoracic surgery	110	2.49	174	3,998
Urology	197	3.14	166	2,522
All surgical procedures	2,634	2.45	160	2,911

Note: The monetary values of surgical fees were converted to US dollars at $1 = ¥150.

### Main results

The natural logarithms of MI values, representing percentage changes in productivity, are presented in Table 3. No statistically significant difference in overall productivity change was observed between the pre- and postreform periods. Productivity changes across all surgical procedures did not differ significantly from 0 in either period, indicating no overall productivity change before or after the reform. Subgroup analyses revealed no surgical specialty with a statistically significant difference in productivity change between the two periods.

**Table 3.** Productivity Change (%).

Specialties	Prereform, mean (SE)	Postreform, mean (SE)
Cardiovascular surgery	−19.8 (2.0)*	−15.4 (2.6)*
Emergency surgery	−5.0 (16.3)	−6.6 (15.6)
General surgery	−16.5 (18.5)	−6.1 (6.3)
Neurosurgery	−9.1 (44.7)	−44.3 (17.4)*
Obstetrics and gynecology	+10.6 (11.6)	+15.5 (25.2)
Orthopedics	+12.8 (10.1)	−7.5 (6.8)
Otolaryngology	−22.7 (8.7)*	+2.1 (8.7)
Plastic surgery	−6.4 (14.5)	+70.5 (55.0)
Thoracic surgery	+28.5 (13.4)*	−4.4 (3.8)
Urology	−9.5 (12.2)	−2.0 (18.8)
All surgical procedures	−3.5 (6.5)	+0.2 (7.1)

Note: There was no statistically significant difference between “prereform” and “postreform.” *Indicates that the value is significantly different from 0 (p < 0.05). SE: standard error.

### Other analyses

The natural logarithms of EC values, representing percentage changes in efficiency, are shown in Table 4. A statistically significant difference in overall EC was observed between the pre- and postreform periods (p = 0.0004). EC across all surgical procedures differed significantly from 0 in the prereform period. Subgroup analyses demonstrated that otolaryngology was the only specialty with a significant difference in EC between the two periods.

**Table 4.** Efficiency Change (%).

Specialties	Prereform, mean (SE)	Postreform, mean (SE)
Cardiovascular surgery	−1.6 (7.3)	−14.8 (1.2)*
Emergency surgery	−39.3 (30.8)	+5.7 (16.7)
General surgery	−43.7 (26.1)	+4.7 (5.4)
Neurosurgery	−39.1 (59.0)	−36.4 (19.8)
Obstetrics and gynecology	−31.2 (19.7)	+28.6 (28.8)
Orthopedics	−28.0 (15.2)	+6.1 (6.6)
Otolaryngology^†^	−103.7 (36.5)*	+18.1 (11.1)
Plastic surgery	−67.5 (20.9)*	+84.5 (54.8)
Thoracic surgery	+23.9 (5.0)*	+6.4 (3.6)
Urology	−16.6 (27.4)	+12.3 (16.2)
All surgical procedures^†^	−38.2 (9.7)*	+11.8 (7.4)

*Indicates that the value is significantly different from 0 (p < 0.05). ^†^Indicates that there was a statistically significant difference between “prereform” and “postreform” (p < 0.05). SE: standard error.

The natural logarithms of TC values, representing percentage changes in technology, are shown in Table 5. Overall TC differed significantly between the pre- and postreform periods (p = 0.0000). TC across all surgical procedures differed significantly from 0 in both periods. Subgroup analyses demonstrated significant differences in TC between the pre- and postreform periods for cardiovascular surgery, general surgery, orthopedics, and plastic surgery (p = 0.029, p = 0.014, p = 0.018, and p = 0.016, respectively).

**Table 5.** Technical Change (%).

Specialties	Prereform, mean (SE)	Postreform, mean (SE)
Cardiovascular surgery*	−18.2 (5.4)^†^	−0.6 (2.4)
Emergency surgery	+34.4 (20.9)	−12.3 (1.7)^†^
General surgery*	+27.1 (12.8)	−10.8 (1.1)^†^
Neurosurgery	+30.0 (18.1)	−6.9 (3.4)^†^
Obstetrics and gynecology	+41.8 (22.3)	−13.1 (4.3)^†^
Orthopedics*	+40.9 (18.6)	−13.6 (1.6)^†^
Otolaryngology	+81.0 (30.0)^†^	−16.0 (5.5)^†^
Plastic surgery*	+61.2 (19.8)^†^	−14.0 (1.8)^†^
Thoracic surgery	+4.6 (12.8)	−10.7 (2.0)^†^
Urology	+7.1 (17.1)	−14.3 (4.7)^†^
All surgical procedures*	+34.7 (6.5)^†^	−11.6 (0.9)^†^

*Indicates that there was no statistically significant difference between “prereform” and “postreform” (p < 0.05). ^†^Indicates that the value is significantly different from 0 (p < 0.05). SE: standard error.

## Discussion

### Key results

The results did not support our hypothesis presented in the Introduction that the workstyle reform for doctors would significantly increase their surgical total factor productivity in the long term. There was no significant difference in surgical productivity changes between before and after the workstyle reform. However, there was a significant increase in EC between the pre- and postreform periods, whereas there was a significant decrease in TC between the pre- and postreform periods. This is the first study to elucidate the long-term effects of workstyle reform for doctors on surgical productivity changes using actual surgical data.

### Interpretation

The reasons underlying these findings could not be determined from this study. Because this was a retrospective observational study, any explanations remain speculative.

The workstyle reform certainly improved surgical efficiency. Efficiency depends on individual surgeons’ efforts. The observed improvement in efficiency may be attributable to reductions in the risk of medical errors and improvements in doctors’ health in the long term. However, the technological frontier significantly regressed following implementation of the workstyle reform. TC reflects shifts in the technological frontier that are not directly related to labor efficiency ^(9)^. Several possible explanations may account for these changes, although they are speculative and not directly supported by the data presented in this study. Surgeons might have hesitated to attempt innovative surgical procedures in anticipation of the workstyle reform, contributing to regression of the technological frontier. In addition, hospital administrators had prepared for implementation of the workstyle reform before April 2024 and might have delayed new technological investments because of anticipated adverse managerial effects of the reform. In fact, several surgical devices were not updated or repaired until the full effects of the reform became apparent. These counterbalancing effects of efficiency and technical changes might have contributed to the lack of overall productivity change.

### Limitations

This study has some methodological limitations. First, we defined the output solely as surgical fees. However, reimbursement does not necessarily reflect surgical complexity, quality, or outcomes. Important aspects such as surgical complexity and postoperative outcomes were not fully incorporated into the analysis. Our selection of inputs and outputs for the MI model might have affected our findings.

Second, we did not examine differences in surgical outcomes. We only excluded surgical procedures in which patients died within 1 month after surgery to maintain a constant quality outcome. Although patient death within 1 month after surgery does not accurately represent surgical outcome quality, it was the only available outcome measure that was common to the various surgeries analyzed in this study ^(14)^.

Finally, our results might have been biased because of our exclusion criteria and the short study periods of 4 months per year over a 4-year period. Because of budgetary and time constraints, we could not collect full-year data. We collected data only for 4 months (June-September) each year, although we attempted to minimize the risk of systematic bias by analyzing all surgical procedures during the same study period across the 4 years. We excluded minor surgeries and those surgeries that were not reimbursed under the surgical payment system or had incomplete records. These exclusions and short study periods may have significantly limited the scope of the analysis.

### Generalizability

Generalization may appear difficult because this study was based on data from a single institution, and institution-specific factors such as staffing patterns, administrative policies, and case mix may have substantially influenced the results. However, there is an advantage to studying surgical productivity within a single institution. Because ancillary services, such as operating room nursing practices, represent significant resource inputs, these factors are held constant within a single hospital. By analyzing surgeons in the same hospital, all surgeons were exposed to the same systemic advantages and disadvantages of these services ^(21)^. Moreover, Teikyo University Hospital is categorized as “A standard,” which imposes the strictest restrictions on doctors’ overtime and therefore reflects the maximum effects of this workstyle reform ^(11)^. Hospitals operating under less strict regulations are unlikely to experience any productivity changes because no productivity changes were observed in a hospital operating under the strictest restrictions. Therefore, our results are generalizable within Japan, although they may not be applicable to other countries.

### Conclusions

No evidence was found to suggest that the workstyle reform for doctors in Japan increased their surgical total factor productivity in the long term. The counterbalancing effects of efficiency and technical changes resulted in no change in overall productivity.

## Article Information

### Author Contributions

Yoshinori Nakata: conceptualization, methodology, investigation, writing – original draft preparation, writing – reviewing and editing, and project administration; Yuichi Watanabe: data curation, software, and formal analysis; Akihiko Ozaki: validation and supervision.

### Conflicts of Interest

None

### Ethics Approval

This study was approved by the Teikyo University Institutional Review Board (approval number: 12-030-4).

## References

[1] Tsutsumi A. Workstyle reform for Japanese doctors. Environ Occup Health Pract. 2020;2(1):1-6.

[2] Yamashiro T, Kumamaru KK, Kido A, et al. Work-style reform and use of information and communication technology among diagnostic radiologists in Japan: results of the 2018 JRS/JCR joint survey. Jpn J Radiol. 2020;38(7):636-42. 10.1007/s11604-020-00941-5 32185671

[3] Lockley SW, Cronin JW, Evans EE, et al. Effect of reducing interns’ weekly work hours on sleep and attentional failures. N Engl J Med. 2004;351(18):1829-37. 10.1056/NEJMoa041404 15509816

[4] Landrigan CP, Rothschild JM, Cronin JW, et al. Effect of reducing interns’ work hours on serious medical errors in intensive care units. N Engl J Med. 2004;351(18):1838-48. 10.1056/NEJMoa041406 15509817

[5] Sim J, Choi Y, Jeong J. Changes in emergency department performance during strike of junior physicians in Korea. Emerg Med Int. 2021;2021:1-7. 10.1155/2021/1786728 PMC8285189 34306757

[6] Nakata Y, Watanabe Y, Ozaki A. Short-term effects of workstyle reform for Japanese doctors on their surgical productivity. Cureus. 2026;18(1):e102095. 10.7759/cureus.102095 PMC12924713 41728446

[7] Cooper WW, Seiford LM, Tone K. Data envelopment analysis: a comprehensive text with models, applications, references and DEA-Solver software. 2^nd^ ed. New York: Springer; 2007. 489 p.

[8] Coelli TJ, Rao DSP, O’Donnell CJ, et al. An introduction to efficiency and productivity analysis. 2^nd^ ed. New York: Springer; 2005. 349 p.

[9] Tone K, Tsutsui M. Chapter 6, Malmquist productivity index model. In: Tone K, editor. Advances in DEA theory and applications: with extensions to forecasting models. Hoboken (NJ): John Wiley & Sons; 2017. p. 40-56.

[10] The Strengthening the Reporting of Observational Studies in Epidemiology (STROBE) Statement: guidelines for reporting observational studies [Internet]. Equator Network. 2025 [cited 2025 Dec 1]. Available from: https://www.equator-network.org/reporting-guidelines/strobe/

[11] Workstyle reform for Japanese doctors [Internet]. Ministry of Health, Labour and Welfare. 2025 [cited 2025 Dec 1]. Japanese. https://www.mhlw.go.jp/stf/seisakunitsuite/bunya/kenkou_iryou/iryou/ishi-hatarakikata_34355.html

[12] Social Insurance Institute (Shakaihoken Kenkyuusho). Interpretation of medical reimbursement scales, April 2022 (Ikatensuuhyou no Kaishaku). Tokyo, Japan: Social Insurance Institute; 2022. Japanese.

[13] Social Insurance Institute (Shakaihoken Kenkyuusho). Interpretation of medical reimbursement scales, April 2024 (Ikatensuuhyou no Kaishaku). Tokyo, Japan: Social Insurance Institute; 2024. Japanese.

[14] Nakata Y, Watanabe Y, Ozaki A. Surgical productivity did not suffer despite the states of emergency against the COVID-19 pandemic in Japan: a retrospective observational study. BMC Health Serv Res. 2022;22(1):1267. 10.1186/s12913-022-08669-w PMC9581545 36261820

[15] Nakata Y, Watanabe Y, Otake H, et al. Productivity change of surgeons in an academic year. J Surg Educ. 2015;72(1):128-34. 10.1016/j.jsurg.2014.06.022 25127451

[16] Thanassoulis E, Portela MCS, Despic O. Chapter 3, Data envelopment analysis: the mathematical programming approach to efficiency analysis. In: Fried HO, Knox Lovell CA, Schmidt SS, editors. The measurement of productive efficiency and productivity growth. New York (NY): Oxford University Press; 2008. p. 251-420.

[17] Fare R, Grosskopf S, Lindgren B, et al. Chapter 13, Productivity developments in Swedish hospitals: a Malmquist output index approach. In: Charnes A, Cooper WW, Lewin AY, Seiford LM, editors. Data envelopment analysis: theory, methodology, and application. New York, NY: Springer; 1994. p. 253-72.

[18] Nakata Y, Watanabe Y, Narimatsu H, et al. Influence of the revision of surgical fee schedule on surgeons’ productivity in Japan: a cohort analysis of 7602 surgical procedures in 2013-2016. Health Serv Manage Res. 2018;31(1):51-6. 10.1177/0951484817740289 29168670

[19] Glantz SA, Slinker BK. Primer of applied regression and analysis of variance. New York (NY): McGraw-Hill; 1990. 777 p.

[20] Fitzmaurice GM, Laird NM, Ware JH. Applied longitudinal analysis. 2^nd^ ed. Hoboken (NJ): John Wiley & Sons; 2011. 752 p.

[21] Chilingerian JA. Chapter 19, Exploring why some physicians’ hospital practices are more efficient: taking DEA inside the hospital. In: Charnes A, Cooper WW, Lewin AY, Seiford LM, editors. Data envelopment analysis: theory, methodology, and application. New York (NY): Springer; 1994. p. 167-94.

